# Bronchodilator effect of deep inspiration and bronchoconstriction-triggered cough

**DOI:** 10.1186/1745-9974-5-9

**Published:** 2009-11-20

**Authors:** Noriyuki Ohkura, Masaki Fujimura, Akira Tokuda, Johsuke Hara, Akihiro Hori, Masaru Nishitsuji, Miki Abo, Nobuyuki Katayama

**Affiliations:** 1 Respiratory Medicine, Cellular Transplantation Biology, Kanazawa, University Graduate School of Medical Science, Japan

## Abstract

**Background:**

Cough in the patients with cough variant asthma is triggered by bronchoconstriction, which responds to bronchodilator therapy. Following airway narrowing induced by inhaled methacholine, deep inspiration (DI) causes dilation of the airways in both asthmatic and non-asthmatic subjects. The aim of the present study was to investigate the relationship between bronchodilator effect of DI and bronchoconstriction-triggered cough.

**Methods:**

We measured airway responsiveness to methacholine using partial and full flow-volume curves in 28 healthy adults. The expiratory flow at 40% above residual volume from the full forced vital capacity (MEF_40_) was obtained and the volume was used as the reference volume to determine the isovolume flow from the partial curve (PEF_40_). Coughs were counted for 32 min during and following the inhalation of methacholine at the provocative concentration which produced a 20% fall or more in FEV_1_from the post-saline value (PC_20_-FEV_1_). The bronchodilator effect of DI on bronchoconstriction induced by methacholine at the PC_20_-FEV_1 _concentration was expressed as the ratio of (MEF_40_-PEF_40_)/PEF_40 _(DI index).

**Results:**

The number of coughs for 32 min during and following the inhalation of PC_20_-FEV_1 _concentration of methacholine was 39.3 ± 29.7 (mean ± SD)/32 min. The number of coughs during and following the inhalation was correlated with DI index (r = 0.57, p = 0.0015), but not with PC_20_-FEV_1 _or change in FEV_1 _or PEF_40 _by inhalation of the PC_20_-FEV_1 _concentration of methacholine.

**Conclusion:**

We found that methacholine-induced cough was associated with the bronchodilator effect of DI on methacholine induced-bronchoconstriction in normal subjects.

## Introduction

Cough is a common and distressing symptom. Eosinophilic airway disorders such as bronchial asthma (BA), cough variant asthma (CVA) [1], eosinophilic bronchitis without asthma [2], and atopic cough (AC) [3] are important causes of chronic non-productive cough. As the mechanism of cough, at least the following two are considered: one is a cough caused by cough reflex hypersensitivity that is relevant to AC, gastroesophageal reflux disease (GERD) [4] and angiotensin-converting enzyme inhibitor-induced cough [5]. Another is a cough triggered by bronchoconstriction in such as CVA and BA, which responds to bronchodilator therapy [1,6].

Capsaicin has achieved widespread use for measurement of cough reflex sensitivity [7]. Several studies suggest that capsaicin-induced cough is mediated by the selective excitation of C-fiber receptors, which have thin non-myelinated vagal afferents, and by the subsequent release of tachykinins. Recently, the C-fiber receptor for capsaicin has been identified as transient receptor potential vanilloid 1 (TRPV1), which is expressed in guinea pigs. TRPV1 mediates cough induced by capsaicin [8]. An increased expression of TRPV1 has also been reported in humans with chronic cough [9]. On the other hand, the mechanism of cough triggered by bronchoconstriction is not clear yet. It has been reported that cough sensitivity to capsaicin or mannitol does not directly correlate to bronchoconstriction in normal and asthmatic subjects [10-12]. Inhalation of methacholine produces bronchoconstriction, and methacholine challenge test is usually done for evaluating bronchial hyperresponsiveness (BHR) [13]. There are few reports concerning cough induced by methacholine inhalation in human, because it is known that methacholine is a bronchoconstrictor, but not an efficacious cough inducer [14].

It is well recognized that the response of the airways to deep inspiration (DI) differs between asthmatic and nonasthmatic subjects. Following airway narrowing induced by inhaled methacholine, DI causes dilation of the airways in both asthmatic and nonasthmatic subjects [15]. However, the magnitude of the bronchodilating effect is usually less in asthmatic than in nonasthmatic subjects. It has been suggested that the inability of DI to overcome bronchoconstriction is a fundamental abnormality of asthma, and could contribute to BHR [15].

The aim of the present study was to investigate the correlation of cough triggered by methacholine-induced bronchoconstriction to airway smooth muscle (ASM) tone and bronchodilating effect of DI on methacholine-induced bronchoconstriction in normal subjects.

## Materials and methods

### Subjects

Twenty-eight normal subjects [2 men and 26 women, mean age 21.5 ± 1.3 (standard deviation, SD) years] were selected from 30 randomly selected Japanese college students who visited our pulmonary function laboratory for actual training of pulmonary function testing including methacholine challenge test using partial and full flow-volume curves. Subjects with a history of wheezing were excluded (Table 1). All subjects gave informed consent before entering the study. The Ethics Committee of Kanazawa University Hospital approved the present study.

**Table 1:** Characteristics of 28 non-asthmatic healthy young adults and values for FVC, FEV_1_, MEF_40_, PEF_40 _and (MEF_40_-PEF_40_)/PEF_40 _ratio at baseline

Gender:	2 men and 26 women
Age:	21.5 ± 1.3 years old
Height:	159.9 ± 5.5 cm
Weight:	51.0 ± 5.6 kg
FVC:	3.46 ± 0.72 L (107.4 ± 13.4% of predicted value)
FEV_1_:	3.20 ± 0.62 L (97.7 ± 11.7% of predicted value)
FEV_1_/FVC ratio:	92.7 ± 4.2%
MEF_40_:	3.56 ± 0.72 L/s
PEF_40_:	3.41 ± 0.82 L/s
(MEF_40_-PEF_40_)/PEF_40 _ratio:	0.074 ± 0.22

### Study Design

**Figure 1:**
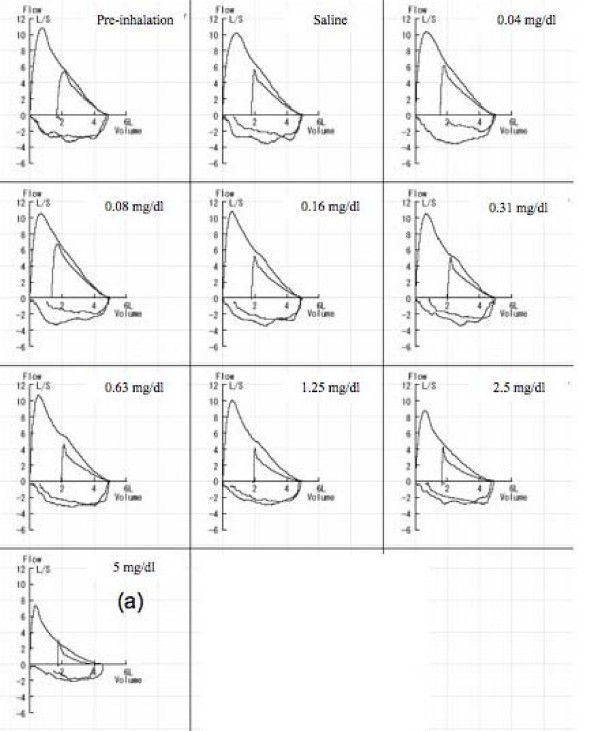
**Study design is shown with an example**. Increasing concentrations of methacholine (0.04, 0.08, 0.16, 0.31, 0.63, 1.25, 2.5, 5, 10, 20, 40, 80, and 160 mg/ml) were inhaled until a fall of 20% or more in FEV_1_(PC_20_-FEV_1_) from the post-saline value occurred. After each concentration of methacholine inhalation, partial and full flow-volume curves were measured. Coughs were counted for 2 min during the inhalation and for 30 min following the inhalation of methacholine at PC_20_-FEV_1_. [(a) In this case].

We measured airway responsiveness to methacholine using partial and full flow-volume curves in 28 normal subjects. Study design is shown in Figure 1. Increasing concentrations of methacholine were inhaled until a fall of 20% or more in FEV_1 _(PC_20_-FEV_1_) from the post-saline value occurred. After each concentration of methacholine inhalation, partial and full flow-volume curves were measured. Coughs were counted for 2 min during the inhalation and for 30 min following the inhalation of methacholine at PC_20_-FEV_1_. After cough count, the bronchoconstriction was subsequently reversed with salbutamol.

### Methacholine inhalation challenge

Methacholine chloride was dissolved in physiologic saline to make solutions of 0.04, 0.08, 0.16, 0.31, 0.63, 1.25, 2.5, 5, 10, 20, 40, 80, and 160 mg/ml. Physiologic saline solution and methacholine were inhaled from a nebulizer (DeVilbiss 646, DeVilbiss Health Care; Somerset, PA) operated by compressed air at 5 l/min. The nebulizer output was 0.28 ml/2 min. Saline solution was inhaled first for 2 min and partial and full flow volume curves were measured using a computerized spirometer (CHESTAC-9800; CHEST; Tokyo, Japan). After confirming that the change in FEV_1 _from the baseline value after inhalation of saline solution was 10% or less, inhalation of methacholine was started. Methacholine was inhaled for 2 min by tidal mouth breathing wearing a nose clip, and this was followed immediately by 3 measurements of partial and full flow-volume curves at 1 min intervals and the curve with the largest forced vital capacity (FVC) was retained for analysis. Subjects avoided to deep inspiration (DI) before the each measurements of partial and full flow-volume curves. Increasing concentrations of methacholine were inhaled until PC_20_-FEV_1_.

### Partial and full flow-volume curves

Partial and full flow-volume curves were measured by the modified method of Fish and co-workers [15]. Flow-volume curve maneuvers were performed in the following manner. After a period of normal tidal breathing subjects momentarily held their breath at slightly above the end-tidal inspiration (approx 60% of FVC) and then forcibly expired to residual volume (RV) level. After reaching the RV level, subjects inspired to total lung capacity (TLC) level as rapidly as possible (this is deep inspiration.) and then immediately performed forced expiration. The expiratory flow at 40% above RV level from the full forced vital capacity (MEF_40_) was obtained and the volume level was used as the reference volume level to determine the isovolume flow from the partial curve (PEF_40_). The levels of end-tidal inspirations were similarly adjusted in all partial flow-volume curves. (Figure 1)

**Figure 2:**
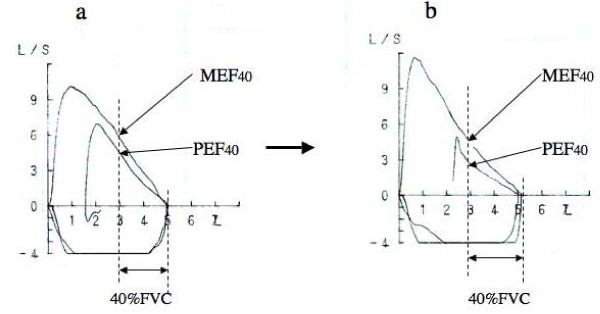
**Examples of partial and full expiratory flow-volume curves before (a) and after (b) inhalation of methacholine in a young woman**. Partial curves were performed from end-tidal inspiration; upon reaching residual volume level, subjects inspired to total lung capacity level and performed the full curve. In this example MEF_40 _was greater than PEF_40 _before methacholine inhalation, and the difference became much more after inhalation of 0.5 mg/ml of methacholine, indicating stronger bronchodilator effect of deep inspiration (DI) on methacholine-induced bronchoconstriction.

The bronchodilating effect of DI on methacholine induced-bronchoconstriction was expressed as the ratio of (MEF_40_-PEF_40_)/PEF_40 _[16] (Figure 2). Especially, the ratio of (MEF_40_-PEF_40_)/PEF_40 _after the inhalation of PC_20_-FEV_1 _concentration of methacholine was defined as DI index. DI index means bronchodilating effect of deep inspiration following measurements of partial flow-volume curves on PC_20_-FEV_1_concentration of methacholine-induced bronchoconstoriction.

### Data analysis

Values of PC_20_-FEV_1_, concentration of methacholine which produced 35% or more fall in MEF_40 _(PC_35_-MEF_40_) and PEF_40_(PC_35_-PEF_40_) were expressed as geometric means with the geometric standard error of the mean (GSEM) expressed as a factor. Values for FVC, FEV_1_, MEF_40_, and PEF_40 _were reported as arithmetic means and standard deviations of the mean (SD). Wilcoxon signed-ranks test was applied to assess the change in the ratio of (MEF_40_-PEF_40_)/PEF_40_. We constructed simple regression models to evaluate the relationship between any pairs of the number of coughs for 32 min during and following the inhalation of PC_20_-FEV_1 _concentration of methacholine, DI index, concentration of inhaled methacholine and changes in FEV_1 _and PEF_40 _by the methacholine inhalation. In all analyses, values of p < 0.05 were considered statistically significant.

## Results

The mean values for FVC, % predicted value of FVC, FEV_1_, % predicted value of FEV_1_, FEV_1_/FVC ratio, MEF_40_, PEF_40_, (MEF_40_-PEF_40_)/PEF_40 _are shown in Table 1. The geometric mean values for PC_20_-FEV_1_, PC_35_-MEF_40_, and PC_35_-PEF_40_, the mean value for percent change in FEV_1_, MEF_40_, and PEF_40 _from the baseline value, DI index and the number of coughs for 32 min during and following the inhalation of PC_20_-FEV_1 _concentration of methacholine are shown in Table 2. In 9 (32.1%) of 28 normal subjects, 20% or more fall in FEV_1 _did not occur at the final concentration of methacholine solution (160 mg/ml), and the PC_20_-FEV_1 _value for these subjects was assumed to be 320 mg/ml for statistical analysis. 35% or more fall in MEF_40 _was not produced by the final concentration of methacholine in 4 (14.2%) of 28 subjects and the PC_35_-MEF_40 _value for these subjects was assumed to be 320 mg/ml for statistical analysis. On the other hand, 35% or more fall in PEF_40_was produced by 160 mg/ml or less of methacholine in all subjects.

**Table 2:** Airway responsiveness to methacholine, DI index and number of coughs for 32 min during and following the inhalationof methacholine

Airway responsiveness	
PC20-FEV1	71.6 (GSEM, 1.28) mg/ml
PC35-MEF40	21.1 (GSEM, 1.28) mg/ml
PC35-PEF40	6.22 (GSEM, 1.27) mg/ml
After inhalation of PC20-FEV1 concentration of methacholine	
Percent decrease in FEV1 from the baseline value	22.0 ± 9.0%
Percent decrease in MEF40 from the baseline value	53.4 ± 20.8%
Percent decrease in PEF40 from the baseline value	63.0 ± 18.2%
(MEF40-PEF40)/PEF40 ratio (DI index)	0.41 ± 0.39
Number of coughs	39.3 ± 29.7/32 min

**Figure 3:**
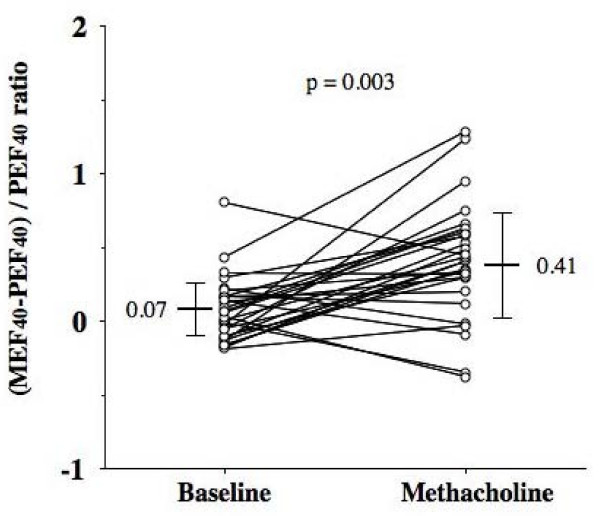
**Deep inspiration (DI) effect on bronchomotor tone before and after inhalation of methacholine at individual concentration causing 20% or more fall in FEV_1_**. (MEF_40_-PEF_40_)/PEF_40 _ratio was calculated as an index of DI effect on bronchomotor tone.

The mean value for the ratio of (MEF_40_-PEF_40_)/PEF_40 _following inhalation of methacholine at individual concentration causing 20% or more fall in FEV_1 _(DI index) was significantly increased from the baseline value. (0.41 ± 0.39 from 0.074 ± 0.22, p = 0.003) [Figure 3].

**Figure 4:**
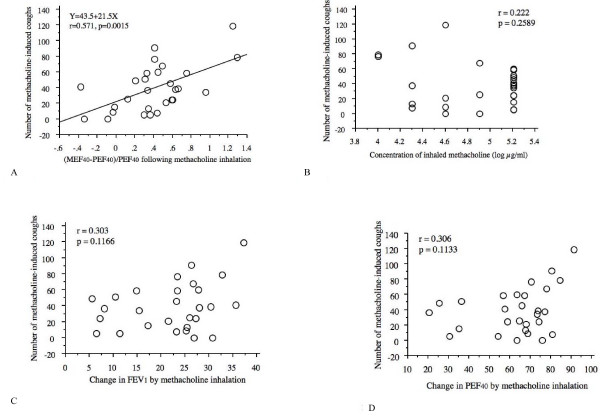
**Correlation of number of methacholine-induced cough with deep inspiration (DI) effect on methacholine-induced bronchoconstriction (a), concentration of inhaled methacholine (b), intensity of methacholine-induced bronchoconstriction assessed by FEV1 (c) and intensity of methacholine-induced bronchoconstriction assessed by PEF40 (d)**. Concentration of methacholine in this case means the concentration causing 20% or more fall in FEV_1 _in individual subjects.

The number of coughs for 32 min during and following the inhalation of PC_20_-FEV_1 _concentration of methacholine was 39.3 ± 29.7/32 min. As shown in Figure 4, the number of coughs during and following the inhalation was significantly correlated with DI index (r = 0.57, p = 0.0015), but not with PC_20_-FEV_1 _concentration of methacholine (r = 0.22, p = 0.26), or change in FEV_1 _(r = 0.30, p = 0.12) or PEF_40 _(r = 0.31, p = 0.11) by inhalation of the PC_20_-FEV_1 _concentration of methacholine.

## Discussion

In this study, we measured airway responsiveness to methacholine using partial and full flow-volume curves and coughs caused by the inhalation of PC_20_-FEV_1 _concentration of methacholine in 28 normal subjects. The bronchodilating effect of DI on methacholine induced-bronchoconstriction was evaluated using the ratio of (MEF_40_-PEF_40_)/PEF_40_. We found that methacholine-induced cough was associated with the bronchodilating effect of DI on methacholine induced-bronchoconstriction, but not with PC_20_-FEV_1 _concentration of inhaled methacholine or the provocation-induced decrease in FEV_1 _in normal subjects. We, therefore, suggest that cough triggered by bronchoconstriction is regulated by protective response against bronchoconstriction, but not by the magnitude of bronchoconstriction. Canning et al. [17] reported in anesthetized guinea pigs that the administration of methacholine via the pulmonary artery caused bronchoconstriction and vigorously activated rapidly adapting receptors.

DI can affect acute airway narrowing by following two mechanisms: bronchoprotection and bronchodilation. Scichilone and coworkers [18] studied on bronchoprotection and bronchodilation in normal subjects. Wan and coworkers [19] studied on these mechanisms in an *in vivo *preparation of guinea pig airway smooth muscle (ASM). Bronchoprotection was defined in these studies as the reduction in bronchoconstriction resulting from act of DI before the ASM has been stimulated to constrict [19] and bronchodilation as the reduction in bronchoconstriction after ASM activation by spasmogen. Scichilone and coworkers reported that the bronchoprotective effects of DI were stronger than the bronchodilating effects and also that bronchoprotection was absent in asthmatics [18,20]. The effect of stretch on ASM preparations was found to be consistent with the change observed in *in vivo *studies [19], which suggests that the effects of DI on airway narrowing are indeed mediated by its action on ASM.

It has been shown that in asthmatics there is impaired dilatation of the airways in response to stretch [15,21]. This is based on the differences in the response of asthmatic subjects to DI compared with normal subjects. Fish and associates [15] measured changes in airway conductance and partial flow-volume curves in response to methacholine inhalation in asthmatics and patients with allergic rhinitis before and after DI. Although asthmatics showed greater airway narrowing as assessed by either measurement, the difference between asthmatic and rhinitis patients was much less prior to than after DI. They suggested that failure of bronchodilating effect of DI was an important cause of airway hyperresponsiveness in asthma.

In this study, the number of coughs following methacholine inhalation was not correlated with concentration of inhaled methacholine causing a 20% or more fall in FEV_1_, or the methacholine-induced change in FEV_1 _or PEF_40_. Irwin and coworkers [22] studied to determine whether any features of positive results of methacholine inhalation challenge or the results of a one-week trial of inhaled beta-agonist therapy were helpful in determining whether the cough was due to asthma. They concluded that the results of airway responsiveness to methacholine could not predict the response of cough to asthma therapy. These results suggest that severity of cough due to bronchoconstriction does not necessarily consist with BHR. It seems to be clinically important for the treatment strategy of chronic cough [23] to distinguish between cough caused by cough reflex hypersensitivity and cough triggered by bronchoconstriction, because the former doesn't react to the bronchodilator at all, and the latter reacts to the bronchodilator. It is also possible that the mechanism of cough is complex, with contributions from airway inflammation, a heightened cough reflex, and bronchoconstriction.

In conclusion, this is the report concerning cough triggered by methacholine-induced bronchoconstriction in humans. We found that bronchoconstriction-triggered cough was associated with the bronchodilating effect of DI on the induced-bronchoconstriction, but not with PC_20_-FEV_1 _concentration of inhaled methacholine or methacholine-induced decrease in FEV_1 _in normal subjects. Further studies are needed to investigate the relationship among cough triggered by methacholine-induced bronchoconstriction, ASM tone and bronchodilating effect of DI on the methacholine-induced bronchoconstriction in patients with CVA in comparison with typical asthma, atopic cough and so on.

## Conflict of interests Statement

The authors declare that they have no competing interests.

## Authors' contributions

NO recruited the subjects, performed the data collecting and draft the manuscript. MF conceived the study, contributed to its design, data acquisition, data interpretation, and review and correction of the manuscript. AT performed the statistical analysis and data interpretation. JH participated in data acquisition. AH participated in data acquisition. MN participated in data acquisition. MA contributed to data interpretation. NK contributed to data interpretation. All authors have given final approval of the version to be published.
